# Expression, Distribution and Function of the Transient Receptor Potential Vanilloid Type 1 (TRPV1) in Endometrial Cancer

**DOI:** 10.3390/ijms26073104

**Published:** 2025-03-27

**Authors:** Thangesweran Ayakannu, Anthony H. Taylor, Justin C. Konje

**Affiliations:** 1 Endocannabinoid Research Group, Reproductive Sciences Section, Department of Cancer Studies and Molecular Medicine, University of Leicester, Leicester LE1 7RH, UK; superdoc.at@gmail.com (A.H.T.); jck4@leicester.ac.uk (J.C.K.); 2 Obstetrics & Gynaecology Centre of Excellence, Sunway Medical Centre, Petaling Jaya 47500, Malaysia; 3 Division of Obstetrics & Gynaecology, Department of Clinical Medicine and Surgery, Sunway University, Petaling Jaya 47500, Malaysia; 4 Department of Molecular and Cell Biology, University of Leicester, Leicester LE1 7RH, UK; 5 Department of Health Sciences, University of Leicester, Leicester LE1 7RH, UK

**Keywords:** anandamide, apoptosis, cell growth, endocannabinoid, endometrial cancer, vanilloid receptor

## Abstract

The transient receptor potential vanilloid 1 receptor (TRPV1) is a calcium-sensitive membrane receptor activated by capsaicin and the endocannabinoid, anandamide (AEA). Once activated in vitro, endometrial cancer (EC) cell growth appears to be inhibited through increased apoptosis, but the mechanism remains unclear. Our aim was to investigate the expression and distribution of TRPV1 in normal and cancerous endometria and to determine the precise in vitro mechanism of decreased EC cellular growth. TRPV1 expression in patients with endometrial carcinoma (15 Type 1 EC, six Type 2 EC) and six normal patients (atrophic endometria) was assessed using quantitative RT-PCR and immunohistochemistry (IHC). Additionally, immunohistochemical staining for the proliferation marker Ki-67, the pro-apoptotic marker BAX and the anti-apoptotic marker Bcl-2 were explored. TRPV1 transcript (*p* = 0.0054) and immunoreactive protein (*p* < 0.0001) levels were significantly reduced in all EC tissues when compared to control (atrophic) endometria. The almost 50% reduction in TRPV1 transcript levels was mirrored by an almost complete loss of immunoreactive TRPV1 protein. The increased proliferation (Ki-67) of EC tissues correlated with the expression of mutated BAX and inversely correlated to Bcl-2, but only in Type 2 EC samples. In vitro, AEA caused a decrease in Ishikawa cell numbers, whilst capsaicin did not, suggesting the anti-proliferative effect of AEA in EC cells is not via the TRPV1 receptor. In conclusion, the loss of TRPV1 expression in vivo plays a role in the aetiopathogenesis of EC. Activation of cells by AEA also probably promotes EC cell loss through a pro-apoptotic mechanism not involving TRPV1.

## 1. Introduction

Globally, endometrial cancer (EC) has become the most common female gynaecological cancer [1]. In 2020, more than 417,000 new cases were diagnosed and more than 97,000 women died globally from EC [2]. The American Cancer Society estimated that between 63,000 and 69,000 new cases of EC will have been diagnosed in the USA alone and that as many as 12,550 women died from EC in 2022 [3]. The incidence of EC is increasing, becoming a public health disease causing high individual and socio-economical disease burdens [1]. Furthermore, by 2030, the incidence of EC in the USA is projected to increase by 55% above the number of cases diagnosed in 2010 [4]. It is the most common gynaecological malignancy in the USA and the only gynaecological cancer with increasing incidence and mortality [5]. This increasing incidence is attributable mainly to excess oestrogen unopposed by progesterone, increased life expectancy, decreased number of pregnancies, early age at menarche and late-onset menopause, or tamoxifen use for breast cancer and mainly affects post-menopausal women (>55 years old). The other major factor is the presence of metabolic syndrome or its individual components such as obesity, diabetes, hypertension and life-style factors [6].

The endocannabinoid system (ECS) is a complex lipid based system that consists of several ligands, such as *N*-arachidonoylethanolamine (also known as anandamide [AEA]) [7], 2-arachidonoylglycerol (2-AG), *N*-oleoylethanolamide (OEA) and *N*-palmitoylethanolamide (PEA); regulatory enzymes, such as fatty acid amide hydrolase (FAAH) and N-acyl phosphatidylethanolamine-specific phospholipase D (NAPE-PLD; [8,9]); and receptors such as cannabinoid receptor 1 (CB1; first cloned by Devane et al. in 1988 [7]), cannabinoid receptor 2 (CB2; first cloned by Munro et al. in 1993 [10]), GPR55 (first cloned by Sawzargo et al. in 1999 [11]) and transient receptor potential vanilloid type 1 (TRPV1) that was first cloned by Caterina et al. in 1997 [12]. The ECS includes ligands, enzymes and receptors, all of which have been demonstrated to be present in the endometrium, and the ECS is modulated in both oestrogen-dependent (Type 1) and oestrogen-independent (Type 2) EC [8,13,14,15] and in endometrial cancer cell lines [16]. The levels of certain endocannabinoid ligands (AEA and OEA) were reported to be higher in the plasma and endometria of women with EC when compared to non-cancer controls [14,17]. Moreover, other ligands such as PEA also show increased levels in the endometrium of EC patients. The response of cancers such as those of the bladder, pancreas, small intestine and prostate, as well as keratinocyte and neuroblastoma cell lines [18] to endocannabinoid-like ligands, occurs in a non-CB1/CB2-dependent manner [9]. This indicates the existence of other cellular or receptors targets through which endocannabinoids exert their effects in these types of tumours [19]. Subsequently, we and others have demonstrated that the expression of both classical cannabinoid receptors (CB1 and CB2) are significantly lower in EC compared to non-cancerous tissue [13,15,20]. These observations raise an important question: If the concentrations of the ligands increase while their cognate receptor expressions decrease in patients with EC, how do these ligands influence EC cell growth or death? The potential answer lies in the involvement of non-classical cannabinoid receptors (i.e., TRPV1 for AEA) [21], as has been demonstrated in endometrial cancer cell lines [16] and in several other cancers [22,23,24,25,26,27], thereby affecting the balance between cell growth and apoptosis (programmed cell death). We therefore reasoned that similar changes might also exist in EC.

Our aim in the present study was to measure the expression and distribution of TRPV1 at both the transcript (mRNA) and protein (immunohistochemical) level in patients with either Type 1 or Type 2 EC, compared to a control cohort (atrophic endometrium). A second aim was to determine if AEA or a TRPV1 agonist have similar or disparate effects on EC cell survival, by comparing the effects of these agonists on endometrial cancer cell survival in vitro. The final aim was to determine if the growth inhibitory effect of AEA in patients with EC is due to decreased proliferation or anti-apoptotic activities or increased pro-apoptotic activity, as has been demonstrated in cell lines [16]. Hence, the transcript and the protein expression levels of TRPV1 were compared between atrophic and EC tissues and their relative levels correlated with the markers of cellular proliferation and apoptosis, and with the expression patterns of other protein components of the ECS [8,13,20,28].

## 2. Results

### 2.1. Patient Characteristics

Patient details, the type of tissue biopsy produced, their ages and BMIs are shown in Table 1. Although there appeared to be subtle differences in ages and BMIs between the endometrial cancer patients and controls, these differences were not statistically different. All participants were post-menopausal, and these participants were studied because they are the largest group of women (by age) affected by EC. The tissues were classified (and dissected free of surrounding tissue) by an experienced histopathologist using the FIGO classification system [29]. Histological examination revealed the control group endometria were all atrophic.

### 2.2. TRPV1 Transcript Levels

**Figure 1:**
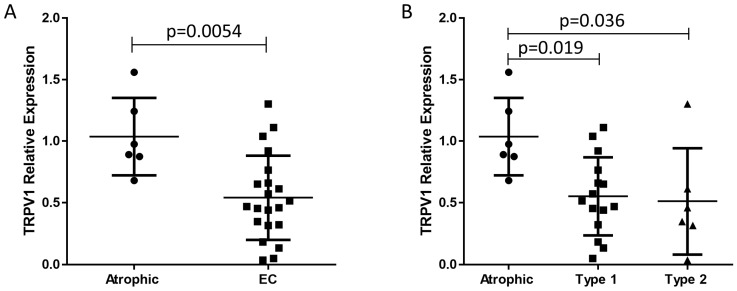
Transcript levels for TRPV1 in endometrial cancer samples. Panel (**A**): *TRPV1* transcript levels were significantly (*p* = 0.0054) lower in the EC tissue (closed circles) [0.468 (0.318–0.712)]; [median ± IQR]) when compared to that of atrophic tissues (closed squares) [0.933 (0.825–1.322)]. Panel (**B**): Sub-analysis of the two types of EC revealed that TRPV1 transcript levels were significantly lower in Type 1 EC than in atrophic samples [0.514 (0.320–0.764)] vs. [0.933 (0.825–1.322)]; [median (IQR)], respectively. In addition, a reduction in TRPV1 transcript levels was observed in Type 2 EC (closed triangles) [0.402 (0.245–0.785)], *p* < 0.05, when compared that of the control (atrophic) group. The Ct values from the RT-qPCR analyses were normalised against the geometric mean of the Ct values for the transcript levels of *IPO8*, *MRPL19* and *PPIA*, as described in [20].

The relative transcript TRPV1 levels in the atrophic and malignant endometria are shown in Figure 1. The transcript levels for TRPV1 were significantly lower in EC, with TRPV1 levels being 1.92-fold higher in the atrophic samples. Additionally, sub-analysis revealed significantly lower levels of TRPV1 transcript in both Type 1 (0.53-fold) and Type 2 (0.49-fold) EC samples compared to atrophic endometrium. However, upon more detailed examination, the lower TRPV1 transcript levels across different tumour grades did not demonstrate statistical significance within individual groups.

### 2.3. Identification and Location of TRPV1 Protein in EC

**Figure 2:**
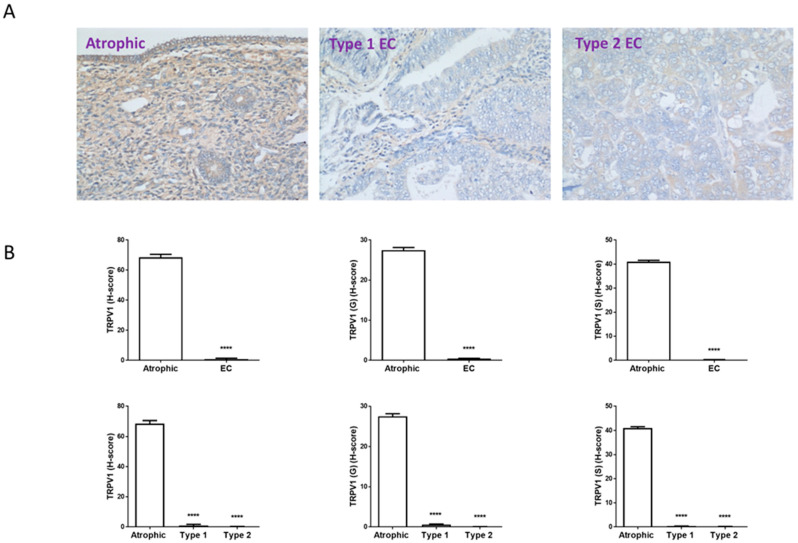
TRPV1 immunohistochemistry staining patterns. Panel (**A**) shows brown (DAB) staining for TRPV1 in representative sections of control (Atrophic), Type 1 and Type 2 endometrial cancer (EC) tissues. Images were captured at 200× magnification. Staining of both stromal and glandular and luminal epithelial tissue is noted in the control tissue, with slightly higher intensity staining within the stromal compartment. By contrast, overall staining levels in the EC tissues were reduced when compared to the control tissue. Panel (**B**) shows the histomorphometric analyses for the whole tissue, for the glands (G) alone or the stroma (S) alone. The histoscore (H-score) was significantly reduced in the entire tissue, glands and stroma (top set of graphs) when compared to the atrophic control (**** *p* < 0.0001; Student’s *t*-test). Sub-type analysis of the Type 1 and Type 2 EC samples indicated that overall staining H-score levels in both types of EC tissues were reduced when compared to the control tissue (lower set of graphs), regardless of whether the entire tissue, glands and stroma when compared to the atrophic control (**** *p* < 0.0001; one-way ANOVA with Tukey’s HSD test).

Figure 2 presents the IHC staining patterns of TRPV1 using target-specific antibodies (references in Supplemental Table S1) in both atrophic and EC tissues. Following the optimisation of TRPV1 antibody (Supplemental Figure S1), all the studies were undertaken in a single run to minimise variations. Within the study group, TRPV1 staining appeared more intense in atrophic compared to EC samples and was observed in both the stroma and the glands.

In atrophic tissue, TRPV1 immunoreactivity was uniformly demonstrated in the cytoplasm of both glandular epithelial and stroma cells. Despite the limited presence of glands in the atrophic tissue, TRPV1 protein was evident, with the strongest staining on the luminal surface (Figure 2A). Additionally, the stroma exhibited uniform staining, with staining apparent throughout the cells. In grade 1 EC, the staining was more concentrated and predominantly associated within the glands rather than the stroma, with the stroma of grade 1 EC tissue exhibiting minimal staining (Figure 2A). Conversely, in grade 2 EC, staining was more prominent in the stroma more than in the glands. In grade 3 EC, the staining was notably faint in both the glands and the stroma. In Type 2 EC, staining was more cytoplasmic and diffuse, with slightly increased immunoreactivity observed in the glands that were scattered throughout the section (Figure 2A); staining in stromal cells was minimal. These staining patterns underwent histomorphometric analyses (Figure 2B). Staining of the entire tissue (left panels) or the glands alone (middle panels) or the stroma alone (right panels) consistently revealed significant reduction in immunoreactive TRPV1 protein levels in the EC samples compared to atrophic control tissues. These observations mirror the transcript data shown in Figure 1. Notably, the levels of TRPV1 protein observed in Type 1 EC tissues were comparable to those in Type 2 EC tissue.

### 2.4. Ki-67, BAX and Bcl-2 Staining Patterns

**Figure 3:**
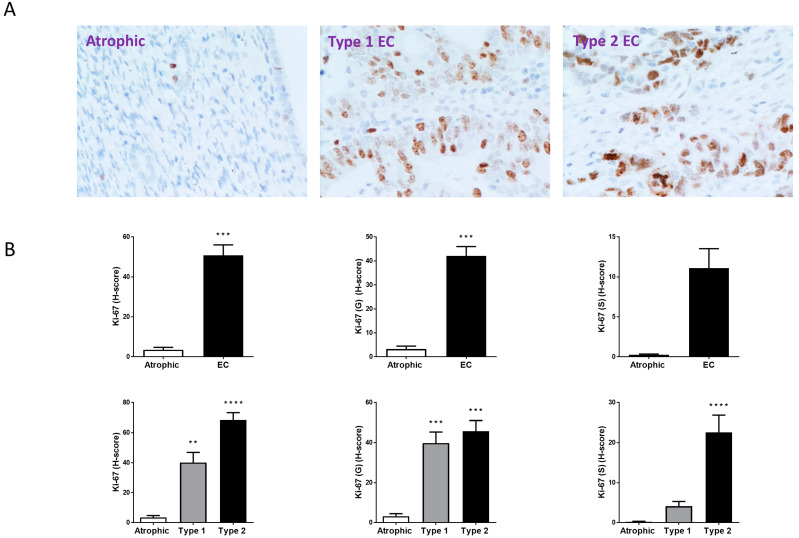
Ki-67 immunohistochemistry staining patterns. Panel (**A**) shows brown (DAB) staining for Ki-67 in representative sections of control (Atrophic), Type 1 and Type 2 endometrial cancer (EC) tissues. Images were captured at 200× magnification. Staining of both stromal and glandular and luminal epithelial tissue is noted in the control tissue, with slightly higher intensity staining of the stromal compartment. By contrast, overall staining levels in the EC tissues were reduced when compared to the control tissue. Panel (**B**) shows the histomorphometric analyses for the whole tissue, for the glands (G) alone or the stroma (S) alone. The histoscore (H-score) was significantly reduced in the entire tissue, glands and stroma (top set of graphs) when compared to the atrophic control *** *p* < 0.001; Student’s *t*-test). Sub-type analysis of the Type 1 and Type 2 EC samples indicated that overall staining H-score levels in both types of EC tissues were reduced when compared to the control tissue (lower set of graphs), regardless of whether the entire tissue, glands and stroma when compared to the atrophic control (** *p* < 0.01; *** *p* < 0.001; **** *p* < 0.0001; one-way ANOVA with Tukey’s HSD test).

**Figure 4:**
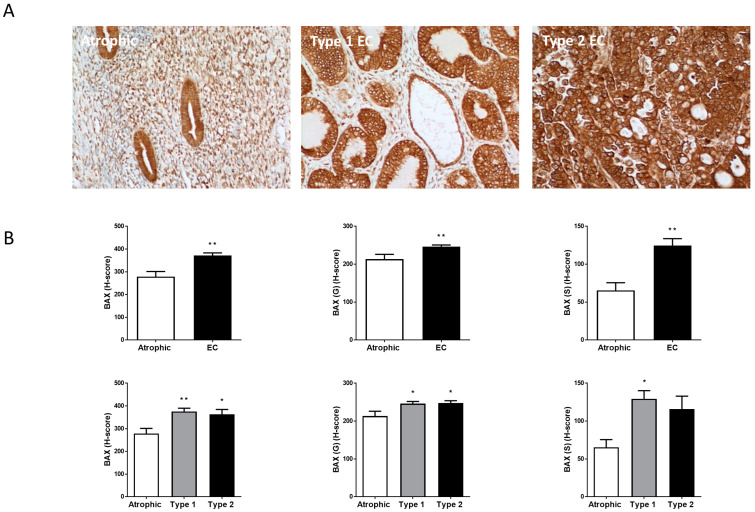
BAX immunohistochemistry staining patterns. Panel (**A**) shows brown (DAB) staining for BAX in representative sections of control (Atrophic), Type 1 and Type 2 endometrial cancer (EC) tissues. Images were captured at 200× magnification. Staining of both stromal and glandular and luminal epithelial tissue is noted in the control tissue, with slightly higher intensity staining of the stromal compartment. By contrast, overall staining levels in the EC tissues were reduced when compared to the control tissue. Panel (**B**) shows the histomorphometric analyses for the whole tissue, for the glands (G) alone or the stroma (S) alone. The histoscore (H-score) was significantly reduced in the entire tissue, glands and stroma (top set of graphs) when compared to the atrophic control (** *p* < 0.01; Student’s *t*-test). Sub-type analysis of the Type 1 and Type 2 EC samples indicated that overall staining H-score levels in both types of EC tissues were reduced when compared to the control tissue (lower set of graphs), regardless of whether the entire tissue, glands and stroma when compared to the atrophic control (* *p* < 0.05; ** *p* < 0.01; one-way ANOVA with Tukey’s HSD test).

**Figure 5:**
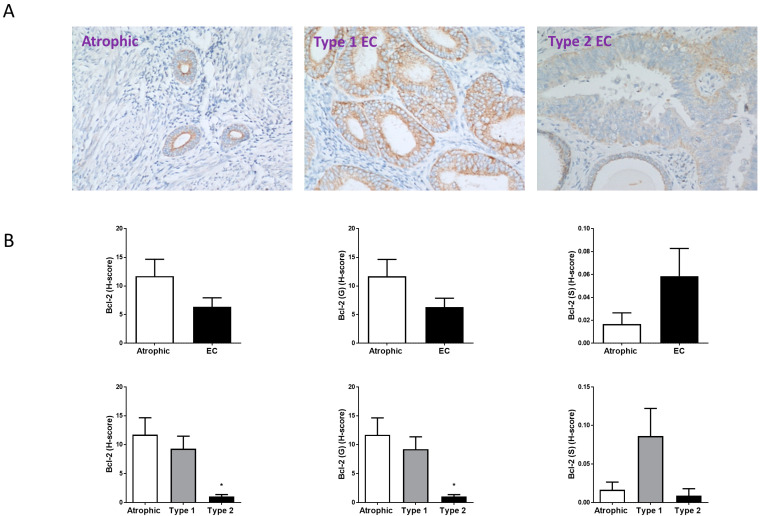
Bcl-2 immunohistochemistry staining patterns. Panel (**A**) shows brown (DAB) staining for Bcl-2 in representative sections of control (Atrophic), Type 1 and Type 2 endometrial cancer (EC) tissues. Images were captured at 200× magnification. Staining of both stromal and glandular and luminal epithelial tissue is noted in the control tissue, with slightly higher intensity staining of the stromal compartment. By contrast, overall staining levels in the EC tissues were reduced when compared to the control tissue. Panel (**B**) shows the histomorphometric analyses for the whole tissue, for the glands (G) alone or the stroma (S) alone. The histoscore (H-score) was unaffected in the entire tissue, glands and stroma (top set of graphs) when compared to the atrophic control * *p* > 0.05; Student’s *t*-test). Sub-type analysis of the Type 1 and Type 2 EC samples indicated that overall staining H-score levels in Type 2 EC tissues were reduced when compared to the control tissue (lower set of graphs), regardless of whether the entire tissue, glands and stroma when compared to the atrophic control (* *p* < 0.05; one-way ANOVA with Tukey’s HSD test).

In order to ascribe a potential function for the TRPV1 receptor in EC, the staining patterns of the cellular proliferation marker Ki-67, the pro-apoptotic marker BAX and the anti-apoptotic marker Bcl-2 were assessed (Figure 3, Figure 4 and Figure 5, respectively). As might be expected for highly proliferative tissues such as Type 1 and Type 2 EC, Ki-67 was significantly increased in EC compared to the atrophic controls. Ki-67 staining was 14.5 ± 8.08 times higher in the EC samples compared to the atrophic control tissues (Figure 3). Although stromal Ki-67 levels were higher in the EC samples, the difference was not significant, whereas in the EC glandular tissue, Ki-67 was significantly (*p* = 0.0001) higher at 12.93 ± 0.37-fold greater than that for the atrophic tissues (Figure 3B). A sub-analysis a differential staining patterns for Ki-67 in the types of cells in Type 1 and Type 2 EC demonstrated slightly higher staining in the Type 2 EC samples than that in Type 1 EC. Sub-analysis revealed that Ki-67 was significantly higher in the glands of Type 1 EC, but not in the stromal cells, whilst it was significantly higher in both glandular and stromal cells of Type 2 EC (Figure 3B).

BAX staining (Figure 4) was the most intense of all the staining patterns observed in endometrial tissue and was found both in the glands and stroma. It was 1.33 ± 0.22-fold higher in EC tissues than in the atrophic control tissues with the greatest difference being in the stromal cells (1.90 ± 0.73-fold higher; Figure 4B). Similar to Ki-67 staining, a differential staining pattern for BAX in the types of cells in Type 1 and Type 2 EC was observed with slightly higher staining in the Type 1 EC than in Type 2 EC. Sub-analysis revealed that BAX was significantly higher in the stromal cells of Type 1 EC, but not in the stromal cells of Type 2 EC, whilst it was significantly higher in the glandular cells of Type 1 and Type 2 EC (Figure 4B).

Bcl-2 staining (Figure 5) was lower than either Ki-67 or BAX in intensity and different to TRPV1 or BAX staining in that immunoreactive Bcl-2 appeared to be lower in EC than in the atrophic control samples (Figure 5). Although there appeared to be more staining in the glandular epithelial cells than in the stroma, the difference was not statistically significant (Figure 5B). The amount of Bcl-2 immunoreactivity in Type 1EC was similar to the atrophic control (both in the glandular and stromal cells) whilst the atrophic control was significantly (*p* = 0.019) 11.01 ± 6.81-fold higher than the Bcl-2 staining in the Type 2 EC tissues (Figure 5B). Although Bcl-2 staining was higher in the stroma of the control and Type 2 EC tissues, the difference was not statistically significant (Figure 5B).

### 2.5. Hallmark of Apoptosis

**Figure 6:**
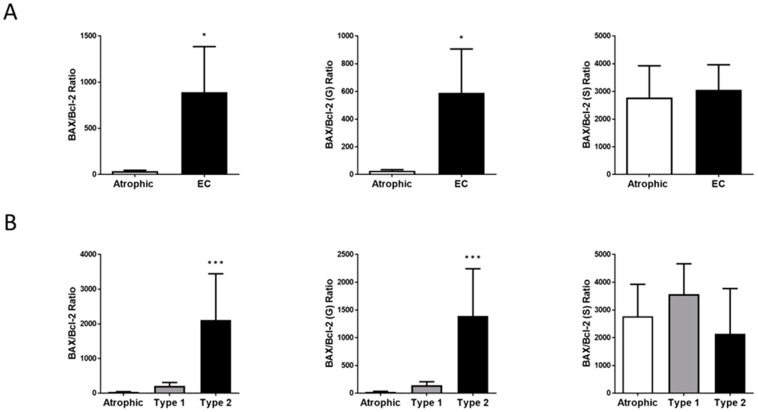
Effect of EC Type on ‘the Hallmark of Apoptosis’. Panel (**A**) shows the relative expression of the pro-apoptotic marker BAX when compared to the expression of the anti-apoptotic marker Bcl-2 in atrophic and endometrial cancer tissues. The BAX:Bcl-2 ratio is higher in EC with the significant effect associated with glandular (G) structures alone (* *p* < 0.05; Student’s *t*-test). Stromal tissues (S) do not contribute to the BAX:Bcl-2 ratio. Sub-group analysis (panel (**B**)) indicated this effect was confined to Type 2 EC where a significant elevation of the apoptosis effects was confined to the glandular (G) tissue (*** *p* < 0.001; one-way ANOVA with Tukey’s HSD test compared to the control atrophic tissue).

The ratio of the H-score patterns for the pro-apoptosis marker BAX to the anti-apoptosis marker Bcl-2 in normal and EC tissues is shown in Figure 6. Using H-score values to generate the ratios (the ‘hallmark of apoptosis’), it was clear that BAX expression was dominant over Bcl-2 expression (Figure 4 and Figure 5) and that is reflected in the BAX to Bcl-2 ratios (Figure 6). This ratio was 25.22 ± 14.01-fold higher in EC than in the control tissues, with the effect due to an increase in pro-apoptotic (BAX staining) and reduced anti-apoptotic (Bcl-2 staining) in the glandular tissue rather than from any changes in the ratio within stromal tissue (Figure 6A). Examination of the two types of EC indicated that the significant event was increased pro-apoptosis in the glands of Type 2 EC tissue, with only a slight (5.97 ± 2.83-fold) but non-significant (*p* = 0.98) rise in Type 1 EC tissues.

### 2.6. Relationships Between TRPV1 Protein Levels with mRNA Levels and That of Other ECS Proteins

**Figure 7:**
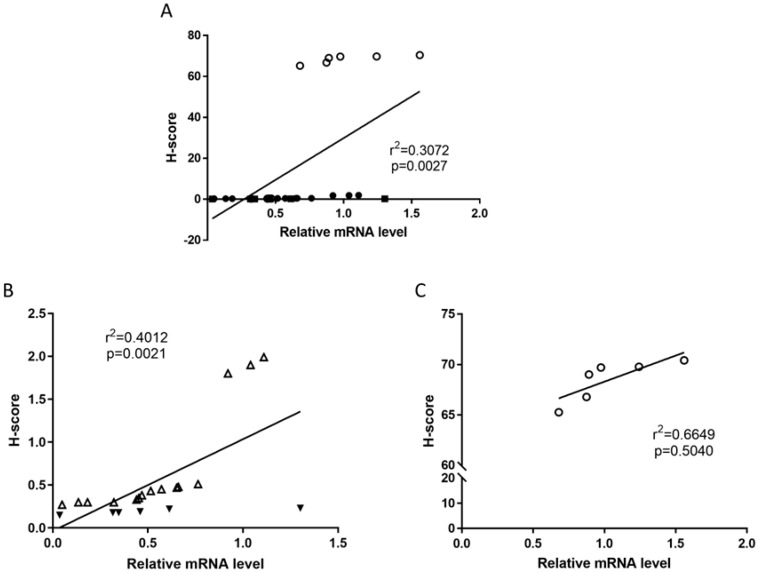
Correlation between transcript and protein levels. Panel (**A**) shows the expected relationship between TRPV1 protein levels determined by H-scores and TRPV1 transcript levels determined by RT-qPCR for the entire set of data. The clear demarcation between H-score values for the atrophic samples (open circles) and the endometrial cancer samples (closed circles) resulted in additional correlations being created for EC alone (Panel (**B**)) and for the atrophic controls alone (Panel (**C**)). In Panel B, the Type 1 EC samples are shown as open triangles and the Type 2 EC samples are shown as closed triangles. The square of the Pearson correlation coefficient (r^2^) is shown together with its attendant *p*-value.

Previously, we reported that the expression of proteins of the ECS do not always correlate with the measured amounts of mRNA that gave rise to those proteins [8]. When the mRNA and protein levels of TRPV1 were plotted for the entire tissue (Figure 7A), it was clear that there were two populations of data: one for the atrophic controls and one for the EC samples. When only the EC tissues were examined (Figure 7B), the three Type 1 samples with the highest TRPV1 mRNA expression levels also had increased TRPV1 protein expression. In contrast, there was a modest but significant relationship between TRPV1 mRNA and protein levels in the control (atrophic) tissues (Figure 7C), indicating that the lack of TRPV1 protein expression is probably important in the pathogenesis of EC.

Examination of possible relationships between the expression of TRPV1 protein and other proteins of the ECS (Supplemental Table S2) indicated no significant linear relationships with any factors in the atrophic control tissues, but significant negative relationships with the levels of Ki-67, CB1, GPR55 and NAPE-PLD proteins in EC. Similarly, there were positive relationships with CB2 and FAAH expression. These data suggest that in EC there are differential changes in several proteins associated with the ECS that are involved in EC pathogenesis. The negative correlation of Ki-67, GPR55 and NAPE-PLD protein expression with TRPV1 expression was only evident in Type 1 EC samples (Supplemental Table S3), whilst the negative correlation with CB1 expression was only evident in Type 2 EC samples. By contrast, the positive correlation between CB2 and FAAH expression with that of TRPV1 was found in both Type 1 and Type 2 EC, suggesting a common cause for the decreased expression of CB1, FAAH and TRPV1 in EC.

### 2.7. Effect of AEA and Capsaicin on EC Cell Survival

**Figure 8:**
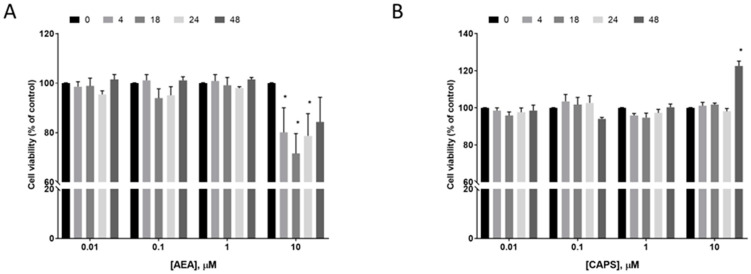
Effect of AEA and capsaicin treatment on Ishikawa cell viability and growth. Panel (**A**) shows the effect of graded doses of AEA on the viability of Ishikawa cells measured by XTT assay at the indicated times (4, 18, 24 or 48 h). Data are shown as mean ± SD of 12 independent measurements. Panel (**B**) shows the effect of equivalent doses of the TRPV1-specific agonist capsaicin (CAPs) on cell viability at the indicated times; * *p* < 0.05 two-way ANOVA with multiple comparisons test.

To determine whether activation of the TRPV1 receptor in EC cells results in reduced cell survival [16], Ishikawa cells were treated with AEA or capsaicin for up to 48 hr (Figure 8). The data indicated that only the highest dose of AEA (10 μM) had a rapid effect on cell numbers (within 4 hr) that persisted throughout the experiment. By contrast, a similar dose of capsaicin (10 μM) had no effect on Ishikawa cell numbers until 48 hr of continuous exposure, when cell numbers increased by 22.6 ± 2.64% over the control (untreated) cell numbers.

## 3. Discussion

Several studies suggested that the expression and actions of the endocannabinoid/vanilloid receptor TRPV1 are altered during the development of tumours and of several cancers [30,31,32,33,34,35,36]. Additionally, it is reported that EC cells in culture enter apoptosis in response to AEA and other ligands through a TRPV1-dependent pathway [16]. Following on from these observations, it was reasoned that similar changes might exist in EC tissues in vivo, but up to the time of writing, this hypothesis had not yet been tested. Hence, the transcript and the protein expression levels of TRPV1 were compared between human atrophic endometria and the two histological types of EC [6]. That TRPV1 expression may also be related to functional components of the ECS and those of cell growth, survival and apoptosis was likewise evaluated.

Surprisingly, TRPV1 transcript (mRNA) levels were found to be highest in the normal atrophic endometrium and greatly reduced in the two types of EC tissue (Figure 1). The level of TRPV1 transcript in normal tissues was not converted efficiently to protein even though significant amounts of TRPV1 protein could be detected in the atrophic control and in normal endometrium and skin (Figure 2 and Supplemental Figure S1). These data suggested that either the antibodies used were not effective in IHC or that in EC there is disconnect between mRNA (transcript) levels and protein expression levels. Accordingly, we performed additional experiments to show the validity of the antibodies in IHC (Supplemental Figure S1) and confirmed TRPV1 staining in the proliferating keratinocytes of the stratum basale of human skin and in the proliferating glandular cells of the endometrium, indicating the antibodies to be useful in IHC and in actively dividing cells. Therefore, our conclusion is that in EC, changes in gene expression at the transcriptome level does not always lead to changes in gene expression at the proteome level (i.e., the remaining 50% of TRPV1 transcript is not efficiently translated into TRPV1 protein). Indeed, this paradigm has recently been discussed as a key point in general carcinogenesis [37] with a disconnect between mRNA levels (transcripts) and protein expression for numerous genes. However, although the relative levels of TRPV1 protein were significantly lower in both the oestrogen-dependent Type 1 EC samples and in the oestrogen-independent Type 2 EC samples, there was enough DAB staining to observe a clear difference in staining patterns in the different grades of Type 1 EC samples. Furthermore, TRPV1 staining in grade 1 EC (the lowest grade of Type 1 EC) was observed to be more in the glands than in the stroma, with the stroma being nearly devoid of staining. By contrast, in grade 2 EC samples (the intermediate grade of Type 1 EC), the staining was observed to be the opposite, with more intense staining being observed in the stromal region than in the glands. Nevertheless, TRPV1 protein levels were significantly lower in all the various grades of Type 1 (1, 2 and 3) and both forms of Type 2 EC (sarcoma and carcinosarcoma). This could be a major cause for concern in our study because our mRNA samples may have been contaminated with stromal elements that could have caused the lack of correlation between transcript and protein levels. However, we were very careful to ensure that only cancer tissue was micro-dissected free during the processing of the biopsies, and so the differential staining in stroma and gland would not be a contributory factor in our analyses. To ensure that was the case, both stromal and glandular staining levels in the histomorphometric analyses were combined to provide a more holistic score akin to that obtained from the RT-qPCR studies, and reasonable correlations between transcript and protein levels were obtained (Figure 7). The alternative hypothesis is that different tumours have different expression levels of transcript and protein and so provide a more variable observation. What is clear, however, from these studies is that TRPV1 protein expression is reduced in both Type 1 and Type 2 EC.

The dysregulation of both ‘classical’ (CB1 and CB2) [13,15,17] and ‘non-classical’ (TRPV1 and GPR55) [21,28] endocannabinoid receptors in EC may provide insights into the variable effects of endocannabinoids observed in different tumour models. For example, in certain tumours like gliomas, that abundantly express cannabinoid receptors, plant-based cannabinoids (phytocannabinoids) have been reported to be more effective in inducing tumour cell death. In other tumour types where cannabinoid receptor expression is absent or low, such as in breast cancer, phytocannabinoids may promote tumour growth and metastasis, or inhibit cytotoxicity, possibly by suppression of the anti-tumour immune response [38]. These observations would align with the reduced expression of CB1, CB2 and TRPV1 receptors in EC, suggesting a potential suppression of the anti-tumour immune response occurs in such individuals [38]. This could be a general phenomenon that extends to other types of cancer, as evidenced by studies in human colorectal cancer where the cells have lower CB1 mRNA and exhibit increased tumour growth [39]. Similarly, reduced cannabinoid receptor expression is associated with an increase in apoptosis in astrocytoma, whereas no apoptosis occurs in astrocytoma cells with high receptor expression [40]. Furthermore, CB1 knockout, but not CB2 knockout APCmin mice (a model of colorectal cancer) have an increased risk of intestinal polyp development, a phenomenon that can be replicated by treatment with CB1 antagonists [39]. These receptor reductions are similar to the findings shown here, where the TRPV1 expression was reduced in the malignant tissues when compared to the benign tissues, suggesting that increased apoptosis may be present in EC.

Conversely, elevated cannabinoid receptor levels have been observed in certain malignancies including prostate cancer, pancreatic cancer and non-Hodgkin lymphoma [31,41], where higher CB1 expression correlates with increased disease severity and poor prognosis in prostate and pancreatic cancer [42,43]. Similarly, TRPV1 is overexpressed in both human prostate cancer [31] and squamous cell carcinoma of the tongue [30]. These observations highlight the variability in cannabinoid or TRPV1 receptor expression patterns across different cancer types, suggesting that receptor expression is likely to be tumour-dependent.

In addition to the effects mediated through cannabinoid receptors, evidence suggests that AEA also can induce cytotoxic effects via a cannabinoid-receptor-independent mechanism [44], with AEA acting as an agonist on TRPV1 receptors. These observations lend support to the hypothesis that loss of CB1 and TRPV1 receptors would result in a loss of the cytotoxic effects of AEA in such tissues. These findings suggest that reduced expression of CB1 and CB2 receptors and the total loss of TRPV1 may play a key role in the pathogenesis of EC. In such cases, endometrial tissues may become less responsive to the repressive effects of circulating and locally produced ligands, particularly AEA [32].

In EC cell lines, the amounts of several proteins also did not correlate well with transcript levels [44]. For example, the Hec50co cells (that represent Type 2 EC tissue) were reported to have higher levels of CB1, CB2 and TRPV1 transcript than that of Ishikawa cells (that represent Type 1 EC tissue), yet in Western blot only the CB2 protein level appeared to be greater [44]. Whether these differences were significant or not is difficult to determine since the authors did not perform those comparisons. Nevertheless, these data suggest that the disconnect between transcript levels and protein in EC tissues continues through into cell lines, supporting the idea that protein expression does not always follow transcript changes in EC (Figure 7) and in other tumours [37].

Because the Ishikawa cell line appears to be a good model of Type 1 EC to study the effects of endo-cannabinoids/vanilloids on cell survival [16], we performed experiments to determine what effects AEA and capsaicin (a TRPV1-specific agonist) have on Type 1 EC cell survival. The data indicated that AEA only inhibited Ishikawa cell survival at the highest concentrations, with the effect significantly maintained for up to 24 h (Figure 8). These data are qualitatively similar to those previously published [16]. When treated with capsaicin for equivalent times and with similar concentrations, Ishikawa cells seemed unaffected and only the highest doses at the longest time-point had any effect on the cells, which showed **increased** cell numbers (Figure 8). These data are contrary to those published by Fonseca et al. [16], where they concluded that AEA and other ligands were acting through the same receptor (TRPV1) to induce apoptosis. If that were the case, then it is reasonable to assume that if AEA is inducing apoptosis through the TRPV1 receptor, then another TRPV1 agonist would have the same effect. The reason that capsaicin did not induce cell death in our study is not known, but points to a possibility that AEA and capsaicin bind to different parts of the TRPV1 receptor, one on the intracellular surface near the T1-TM region [45] and one on the extracellular surface very close to the plasma membrane lipids in the S-region [46,47], respectively. Alternatively, the p53 mutation that Ishikawa cells possess [48] prevented activation of BAX-mediated apoptosis [49]. In other cell types, AEA needs to be transported into the cell in order to have its effect [45], and then it needs to take a different route to its final binding position, which is unaffected by 5′iodoresiniferatoxin (iRTX), which was used in the previous study [16], so the conclusion that different parts of the TRPV1 protein are being targeted by AEA and capsaicin is not unreasonable [50]. Nevertheless, the question remained concerning the cell viability effect of AEA in EC: is it due to increased apoptosis as suggested in a previous study [16] or due to reduced cellular proliferation?

From the studies of Fonseca et al. [16], it was clear that in vitro the effect of AEA was induction of apoptosis. To extend those observations, we examined the expression of cell proliferation and apoptosis markers in the same biopsies used for the TRPV1 protein expression reported herein. These samples were also taken from women who also had tissue and plasma levels of AEA measurements [14], corresponding FAAH and NAPE-PLD H-scores [32], CB1/CB2 H-scores [13] and GPR55 H-scores [28]. Since tissue and plasma AEA levels were increased in these EC patients when compared to the atrophic endometria patients, then this would represent a continuous increased AEA level in in vitro Ishikawa cell experiments.

**Figure 9:**
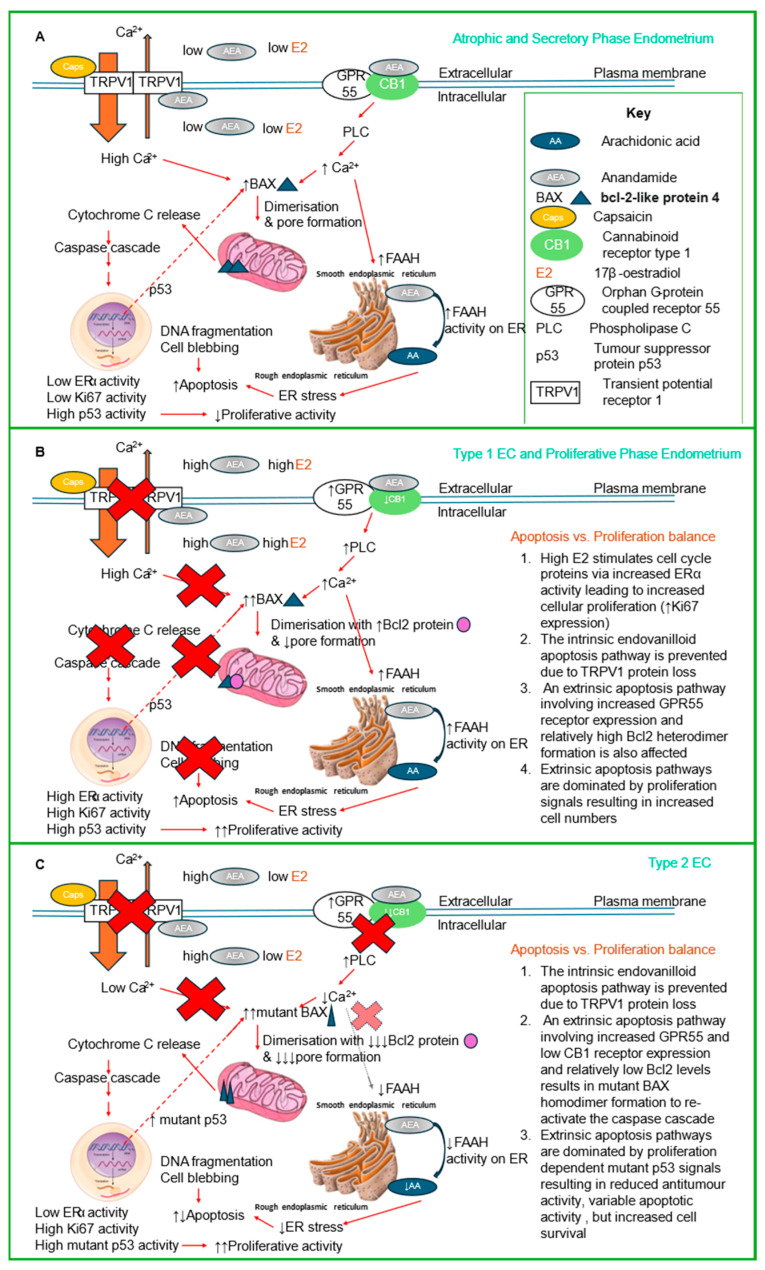
A putative mechanism for TRPV1 and its inactivation in the normal endometrial epithelial cell and in endometrial cancer cell survival. Panel (**A**) shows the key steps that TRPV1 and AEA play in the normal endometrial epithelial cell. The relatively higher concentrations of TRPV1 and low concentrations of AEA activate the TRPV1 in different ways. Capsaicin binding to the extracellular surface of TRPV1 opens the Ca^2+^ ion channel to allow calcium influx. By contrast, AEA binding to the inner surface of TRPV1 induce a small efflux of calcium ions. The resultant higher intracellular Ca^2+^ ion concentrations have two main effects: (1) to allow two BAX molecules to translocate from the cytoplasm to the outer mitochondrial membrane, where bound as a homodimer it opens a pore that allows cytochrome c to escape. The cytochrome c then activates the caspase cascade to initiate DNA fragmentation and cell blebbing that characterises the intrinsic pathway of apoptosis. These cells also have a high tumour suppressor activity in the p53 protein that prevents BAX protein production and a low level of cell cycle proteins. In this proposed model, the relatively low levels of AEA also bind to the relative high levels of the CB1 receptor that homodimerizes with GPR55 producing increased PLC production and through an extrinsic apoptosis pathway results in (2) the mobilisation of FAAH (FAAH-1) to the membrane of the endoplasmic reticulum, where AEA is converted to arachidonic acid (AA), which in turn causes endoplasmic reticulum stress. The stress at the endoplasmic reticulum also leads to cellular apoptosis through various and diverse pathways. The low levels of the steroid hormone 17β-oestradiol (the main inducer of endometrial epithelial cell division) prevent cellular proliferation. So, in this context, the balance of intrinsic and extrinsic apoptosis dominates over cellular proliferation to maintain tissue homeostasis of atrophic and non-proliferative secretory phase endometria of the menstrual cycle. In the case of proliferative endometria and in Type 1 EC (panel (**B**)), the over-riding event is 17β-oestradiol activating its cognate receptor ERα to induce the transcription of cell cycle genes and initiate proliferation. Simultaneously, the TRPV1 receptor disappears, and the intrinsic apoptosis pathway is inhibited (red crosses). The AEA-dependent extrinsic pathway remains active, despite CB1 expression being reduced, resulting in an increased cell proliferation/apoptosis ratio that leads to increased tumorigenesis. In the case of Type 2 EC (panel (**C**)), the key factor involved is mutated proteins. The p53 and BAX proteins are often mutants that have altered activities due to their elongated half-lives. In Type 2 EC, both intrinsic and extrinsic apoptotic pathways are inhibited by the complete loss of TRPV1 and the complete loss of CB1, respectively. The extrinsic pathway is completely inhibited, whilst a small amount of intrinsic activity occurs because mutated p53 allows the formation of excess mutated BAX that remains active at the mitochondrial membrane. Other serum factors (primarily EGF) signal cell cycle and cell proliferation events. In Type 2 EC, cell survival dominates over cell death to allow epithelial cell proliferation and accumulation. Other factors, such as TRPV2, might be involved in the cell migration and metastatic potential prevalent in Type 2 EC. Data collated from this study and [9,12,16,23,28,45,52,57,58,62,63,64,65,66,67,68].

As expected, all of the EC samples showed increased expression of the Ki-67 proliferation marker (Figure 5). Indeed, Ki-67 immunohistochemical staining is often used to classify the molecular subtype of EC present [6,51]. These data were supported by the cell culture experiments performed herein and by Fonseca et al., where Ishikawa cells continue to proliferate in low serum conditions [16] or even in the absence of serum (this study). This suggests that even in the presence of AEA or other TRPV1 agonists, the overarching cell cycle signal remains ‘proliferate’. The demonstration that levels of the pro-apoptotic marker BAX increased in all parts of EC tissue (Figure 4), especially in the glandular epithelial cells, supports the study of Fonseca et al. [16]. Although BAX expression also increased in the stroma but not significantly in the Type 2 EC samples is probably a reflection of the low sample numbers. By contrast, the expression of the anti-apoptotic marker Bcl-2 was statistically unaffected by EC, but sub-analysis of the different types of EC revealed the reason for this was the elevated expression of Bcl-2 in the Type 1 EC glands and stroma (Figure 5). There was a significant reduction in Bcl-2 expression in the Type 2 EC samples, supporting the idea that loss of anti-apoptosis results in the generalised apoptotic effect described in EC [16]. This conclusion was supported by measuring one of the ‘hallmarks of apoptosis’, the BAX:Bcl-2 ratio [52], where it was increased in EC tissue, but only in the glandular epithelial cells and is most acute in the Type 2 EC samples (Figure 6), which is also the most aggressive form of EC that often forms metastatic lesions [6,51]. The BAX:Bcl2 ratio is important because these two proteins work together to control apoptosis, with a homodimer of BAX resulting in pore formation in the outer membranes of mitochondria [53,54], whilst the BAX-Bcl2 heterodimer prevents apoptosis by preventing BAX homodimer binding to the mitochondrial membrane and p53 [54,55], an anti-tumour protein important in preventing cell division [56]. These data suggest that although EC tissue is proliferating, even in the presence of high AEA tissue levels and plasma levels [14], this effect is only partly due to dysfunctional apoptotic signalling and only in the Type 2 EC patient. In patients with Type 1 EC, the elevation of NAEs, such as AEA, is working through a non-intrinsic apoptotic mechanism, possibly utilising a different receptor (Figure 9). Indeed, we recently suggested that in Type 1 EC patients, the key regulator of glandular epithelial cell proliferation is the increased expression of the orphan receptor GPR55, which also binds these ligands [28], possibly through dimerisation with CB1 [57]. The expression of the TRPV1 receptor in EC is evidently significant for understanding the biology of EC tumours, yet further investigation remains imperative. This conclusion is based on the known functions of TRPV1 in cellular homeostasis through the intrinsic apoptotic pathway that involves Ca^2+^ ion influx (Figure 9) and the lack of positive correlation between TRPV1 expression and the BAX:Bcl2 ratio in our patient samples (Supplemental Tables S2 and S3). In the normal situation, activated TRPV1 would result in Ca^2+^ ion influx to affect the intrinsic apoptosis pathway and damage mitochondrial membrane function, resulting from an increase of the BAX:Bcl2 ratio, going on to activate the caspase-dependent apoptosis cascades [58]. In our clinical Type 2 EC samples, the BAX:Bcl2 ratio is inversely correlated with TRPV1 expression, suggesting the intrinsic apoptotic pathway is not active here (Figure 9: [58]). This means that the normal function of BAX (i.e., to bind to mitochondrial membranes to induce apoptosis and to p53 thus prevent anti-tumour events), is no longer functional, thereby allowing cellular proliferation. This was confirmed by the increase in Ki-67 expression (Figure 3), since Ki-67 protein is only produced by cells undergoing active cell division. The observed loss of TRPV1 protein and function in both types of EC directly results in a loss of proliferation control. In the normal proliferative phase endometrium and Type 1 EC, the main cellular proliferation signal is 17β oestradiol, where BAX:Bcl2 ratios are reduced, suggesting that the main regulator of the BAX:Bcl2 ratio in Type 1 EC is 17β oestradiol and not activation of the TRPV1 through an intrinsic apoptosis signal [59]. While there is new evidence supporting the involvement of TRPV1 activation by the vanilloid capsaicin and by the endocannabinoid AEA in regulating cancer cell growth and disease progression in other gynaecological cancers [60], similar studies in EC models suggest that although these agonists might induce apoptosis, an in vivo impact that is unlikely to be substantial in EC [59]. This is primarily due to the considerably lower expression of TRPV1 in EC compared to tissues such as skin, cervix or atrophic or normal proliferating endometrium (see Supplemental Figure S1). Similarly, because the classical cannabinoid receptors CB1 and CB2 also show decreased expression in EC, then AEA is probably activating GPR55 receptors directly instead [28]. Therefore, the elevated levels of plasma and tissue AEA found in patients with EC [14] must affect cell numbers through a non-TRPV1 receptor mediated pathway, perhaps one that involves the TRPV2 receptor, as suggested by Marinelli et al. [61]. In this situation, a mixed Type 1/Type 2 EC cell phenotype would have to be active for AEA to act as an endovanilloid.

In this case, AEA could work co-ordinately with other endocannabinoids (such as 2-AG) through the combination of low TRPV1 activation (to initiate a cell death or apoptosis pathway), whilst 2-AG (or the phytocannabinoid CBD) activates a different part of the apoptosis or autophagy pathways [61]. Clearly, as TRPV2 receptors are expressed in both normal and cancerous tissues, more research in this area is justified. Since TRPV2 has also been shown to be a prognostic marker in Type 2 EC [52], then its expression in Type 1 EC needs to be established. Additionally, since other members of the vanilloid receptor family have not been characterised in EC, then future studies should establish their expression levels and functions especially in EC development and potential in EC patient treatment with TRPV2 receptor-specific antagonists.

In that respect, it is hoped that innovative targeting of TRPV1 receptors [46] or TRPV2 receptors [61] with sub-receptor specific agonists or antagonists (depending on the tissue under investigation) may contribute to a novel tumour-suppressive treatment strategy for EC and other gynaecological cancers. The knowledge that TRPV1 is a ligand-gated Ca^2+^-permeable ion channel, which is usually activated by noxious stimuli such as acid and heat, and is involved in transmission and modulation of pain [69], and may explain why uterine pain is not detected in patients with early-stage EC, when TRPV1 expression is decreasing. Moreover, both AEA and acyl-dopamine are now considered to be endovanilloids because they bind to this receptor [69], and the concomitant increase in tissue AEA production (and other NAEs) we measured [14] may be a reflection of the loss of the receptor in the diseased EC tissue.

A limitation of the findings presented in this study is that we used only one EC cell line. It would be useful to use other cell lines to determine whether our data would be replicated. It is anticipated that our findings with other cell lines would provide robust supportive evidence on the hypothesised mechanism presented herein (Figure 9) or elsewhere [16].

## 4. Materials and Methods

### 4.1. Participants

The women who took part in this study were recruited at the University Hospitals of Leicester (UHL) National Health Service (NHS) Trust. They underwent a hysterectomy due to endometrial carcinoma or benign gynaecological conditions, such as uterine prolapse or leiomyomata. All participants provided signed written informed consent. The study was approved and conducted according to the guidelines of the Leicestershire, Northamptonshire and Rutland Research Ethics Committee (reference number 06/Q2501/49). Participants were excluded if they were currently or had previously been on any form of hormonal treatment (such as hormone replacement therapy or the levonorgestrel intrauterine system) within three months prior to surgery. Women on prescription or recreational drugs were excluded, as well as those with chronic medical conditions (such as diabetes mellitus or hypertension) or those diagnosed with any other type of cancer. Each patient’s age and body mass index at the time of recruitment was recorded.

### 4.2. Sample Collection

The sterile uterus was transported immediately on ice to the histopathology department where a consultant gynaecological histopathologist dissected out two representative biopsies. One biopsy was used for clinical diagnosis by histological methods and immunohistochemistry (IHC), while the other was collected for the measurement of TRPV1 transcript (mRNA) levels. In all cases, the biopsies were washed with phosphate buffered saline (PBS) to remove excess blood and then either immediately stored in RNAlater^®^ (Life Technologies, Paisley, UK) at −80 °C for RNA extraction or in 10% formalin for histological studies. Representative tissue sections (4 μm) were dried onto saline-coated microscope slides and subjected to haematoxylin and eosin (H & E) staining for histological confirmation of disease, which was performed by the consultant gynaecological histopathologist. The tissues were classified using the FIGO classification system [29] into endometrioid (oestrogen-dependent Type 1) and non-endometrioid (oestrogen-independent Type 2) cancer. The Type 1 EC tissues were also classified by grade (1, 2 or 3) and the Type 2 tissues into serous or carcinosarcoma [70]. All the cancer patients were at stage 1 of disease.

### 4.3. RNA Extraction, cDNA Synthesis and Quantitative Real-Time PCR

RNA extractions and cDNA syntheses of the endometrial tissue biopsies (100 mg) were as previously described [20]. The cDNA was stored at −20 °C prior to quantitative real-time PCR (RT-qPCR) analysis. For the RT-qPCR, human TRPV1 (Hs00218912_m1) primers and FAM/MGB dye-labelled probes were used together with three reference genes, MRPL19 (Hs00608519_m1), PPIA (Hs99999904_m1) and IPO8 (Hs00183533_m1) contained on a validated human endogenous control assay TaqMan Array 96-well plates purchased from Applied Biosystems (Life Technologies, Paisley, UK). Previously, we demonstrated these to be the correct endogenous reference genes for atrophic and EC endometrial samples [20]. Included in each run were RT-minus and no template controls (NTC) containing DNAse-free water instead of template mRNA to ensure there was no contamination with exogenous or genomic DNA. No product was synthesised in the NTC and RT-minus samples, confirming the absence of contamination. Each assay had an amplification efficiency of 100% ± 10% (Life Technologies). All the reactions were performed in triplicate and repeated three times to ensure accuracy and reliability of the results. The data were also processed using the relative expression software tool REST2009 ([71]; https://www.gene-quantification.de/rest-xl.html, accessed on 20 January 2025) to confirm the expression levels.

### 4.4. Identification and Tissue Localisation of TRPV1, CB1, CB2, NAPE-PLD, FAAH, Ki-67, BAX and Bcl-2 Protein Expression

Immunolocalisation (IHC) was performed using anti-human TRPV1 antibodies (1:200; rabbit polyclonal anti-TRPV 1 (Alomone labs ACC-030; Har Hotzvim Hi-Tech Park, Jerusalem, Israel), according to the manufacturer’s instructions. The sources and optimised primary antibody dilutions for all the additional proteins are described in Supplemental Table S1. Only detailed descriptions for the TRPV1 immunohistochemistry are described herein because the IHC methodology and results for the other proteins are reported elsewhere [8,13,62,72,73]. Briefly, cut sections dewaxed in xylene and rehydrated through different concentrations of alcohol to de-ionised water (4 min per step), and were exposed to 0.3% H_2_O_2_ in ice-cold ethanol (95%) for 5 min to inhibit endogenous peroxide activity. To remove the peroxide, slides were immersed in Tris-buffered saline (TBS) containing Tween_20_ (TBS-Tween_20_; 0.1%v/v) for 5 min. Normal goat serum (NGS; 5% in TBS; 100 μL/section) for 30 min at room temperature was used to block the tissues further. Avidin solution (4 drops/mL of 1:20 NGS/TBS) was added for 15 min and the slides washed in TBS-Tween_20_ for 5 min twice before the addition of biotin solution (4 drops/mL of 1:20 NGS/TBS) for 15 min. Finally, the slides were drained and incubated with primary antibody (TRPV1) diluted at 1:200 in TBA [1 g BSA/100 mL TBS], whilst non-immune rabbit IgG (1:50 in 1:20 NGS/TBS) was added to IgG control slides. All were then incubated in a humidified chamber at room temperature overnight. Sections were washed in TBS/Tween_20_ in a sandwich box on a magnetic stirrer set at a moderate mixing rate for 20 min before being incubated with biotinylated goat anti-rabbit antibodies diluted 1:400 in TBS for 30 min. The slides were then washed in TBS/Tween_20_ for 20 min and incubated with ABC Elite complex prepared 30 min before use (Vector Labs, Peterborough, UK). After 30 min incubation, slides were washed in TBS/Tween_20_ for 20 min and then stained with 3, 3′-diaminobenzidene (DAB) according to the manufacturer’s instruction (Vector Labs) for 5 min. After washing in tap water for 5 min, the sections were lightly counterstained with Mayer’s haematoxylin (Sigma-Aldrich, Poole, UK) for 1 min and rinsed in tap water until the blue water ran clear. Finally, the slides were dehydrated through graded alcohols, cleared in xylene (3 min/step) and mounted in XAM mounting medium (BDH Chemicals, Lutterworth, UK).

A sample of archival human skin [74] acted as a positive control (Supplemental Figure S1); TRPV1 has previously been shown to present in skin [75]. The TRPV1 antibody was also evaluated in atrophic uterine tissues with an equivalent concentration of IgG, which showed no staining (the negative control) (Supplemental Figure S1). All samples were processed in the same run and repeated twice to avoid any inter-assay variation and ensure reproducibility.

### 4.5. Histomorphometric Analyses

Histomorphometric analysis (H-score) of TRPV1, CB1, CB2, NAPE-PLD, FAAH, Ki-67, BAX and Bcl-2 expression was performed as described [8,13]. Micrographic images were generated in the presence of daylight and medium neutral density filters with the lamp set at 6400 K and captured at 200× or 400× magnification on an Axioplan transmission microscope (Carl Zeiss, Welwyn Garden City, UK) with the aid of a Sony DXC-151P 2/3 inch CCD camera mapping to 768 × 493 pixels (Sony Corp., Yokohama-shi, Japan). Images were analysed using image analysis software (ImageScope version 10.2.2.2319; Aperio Technologies, Inc., Vista, CA, USA) and H-scores recorded. H-scores for the stroma and glands were determined independently and then combined to provide a robust and reproducible H-score for the entire tissue [8,13].

### 4.6. Effect of AEA or Capsaicin on Ishikawa Cell Viability

Ishikawa cells, plated at a cell density of 4 × 10^3^ cells per well in 200 μL of cell culture medium (DMEM:F12 medium containing 10% fetal bovine serum (FBS)) with antibiotics (1% streptomycin and 1U/mL penicillin; all from Gibco, Paisley, UK) to a single 96-well tissue culture plate, were maintained at 37 °C in a humid atmosphere of 5% CO_2_ in air for 24 h. After 24 h, the medium was changed to serum-free DMEM:F12 containing antibiotics, and the cells allowed to grow for another 24 h. At this point, fresh serum-free growth medium containing 1.0 nM to 10 μM of either AEA or the TRPV1-specific agonist, capsaicin (Sigma-Aldrich, Poole, Dorset, UK), was prepared and applied in quadruplicate to the cells. At this point, 50 µL of XTT reagent (Thermofisher Scientific, Leicester, UK) was added and the cultures re-incubated for 4 h. The absorbance at 450 nm and 620 nm was read on a Multiskan Ascent ELISA (MTX Lab Systems Inc., Bradenton, FL, USA) plate reader. The cells were re-incubated for an additional 44 h and re-read on the plate reader at 18, 24 and 48 h. Output data were copied to an Excel file, and the actual XTT absorbance (A_450_-A_620_) determined. The percentage absorbance changes relative to the untreated controls (no drug) were then calculated and reported as % cell viability [37].

### 4.7. Statistical Analysis

Prism version 7.00 for windows (GraphPad Software, San Diego, CA, USA, www.graphpad.com, accessed 20 January 2025) was used to perform the statistical analyses. Normality of the data was determined using the Wilks-Shapiro test and normally distributed data were analysed by unpaired Student’s *t*-test or parametric one–way ANOVA with Tukey’s HSD test. Data that were not normally distributed were expressed as medians and inter-quartile ranges (IQR). Comparison between groups was performed using either the Mann–Whitney U-test or one-way analysis of variance (ANOVA) followed by a multi-comparison test. Tests between variables were considered to be statistically significant when *p* < 0.05. Correlations were performed using Pearson correlation analyses.

## 5. Conclusions

The data presented here thus suggest a possible role for endocannabinoids/endovanilloids in the pathogenesis of EC and although TRPV1 activation holds promise as a potential therapeutic target in certain cancers, it relevance and efficacy in EC warrant further investigation and validation.

## Figures and Tables

**Table 1:** Patients’ ages and BMIs for biopsies analysed by RT-qPCR or immunohistochemistry.

RT-Qpcr *			
Tissue Type	Designation	Age (Years)	BMI (kg/m^2^)
Control	Atrophic (6)	60.67 ± 4.27	26.67 ± 6.50
Type 1 EC	Grade 1 (6)	66.17 ± 16.14	33.50 ± 8.92
	Grade 2 (6)	66.50 ± 10.25	32.00 ± 5.97
	Grade 3 (3)	72.67 ± 12.06	35.33 ± 6.11
Type 2 EC	Serous (3)	59.00 ± 3.46	37.67 ± 2.52
	Carcinosarcoma (3)	50.00 ± 5.00	36.67 ± 6.43
Immunohistochemistry			
Tissue type	Designation	Age (years)	BMI (kg/m^2^)
Control	Atrophic (6)	60.67 ± 4.27	26.67 ± 6.50
Type 1 EC	Grade 1 (6)	62.50 ± 13.90	33.00 ± 8.76
	Grade 2 (6)	65.17 ± 9.86	34.83 ± 5.56
	Grade 3 (6)	66.83 ± 7.88	31.50 ± 3.08
Type 2 EC	Serous (4)	70.25 ± 10.97	33.00 ± 6.83
	Carcinosarcoma (6)	58.33 ± 7.42	35.50 ± 5.99

* Only the biopsies from non-malignant (atrophic) control tissues were used for both RT-qPCR and immunohistochemistry studies; additional EC material was used for the immunohistochemistry studies. The number of samples indicated in parentheses after the designated tissue types. The data are presented as the mean ± SD. The ages and BMIs of samples taken from the different groups were not significantly different from the non-malignant control (atrophic endometrium); one-way ANOVA with Dunnett’s post-test indicated no significant differences in either age or BMI. EC = endometrial cancer.

## Data Availability

The datasets generated or analysed during this study are included in this published article. Additional information is available from the corresponding author upon reasonable request.

## Supplementary Materials

The following supporting information can be downloaded at: https://www.mdpi.com/article/10.3390/ijms26073104/s1.
