# Supplementary material for: Expression, Distribution and Function of the Transient Receptor Potential Vanilloid Type 1 (TRPV1) in Endometrial Cancer
Source: Int J Mol Sci. 2025 Mar 27;26(7):3104. doi: 10.3390/ijms26073104 (PMC11988754; doi:10.3390/ijms26073104)
Supplement: Supplementary file 1 [file ijms-26-03104-s001.zip › Supplemental Figure S1.pdf]

Supplemental file s1

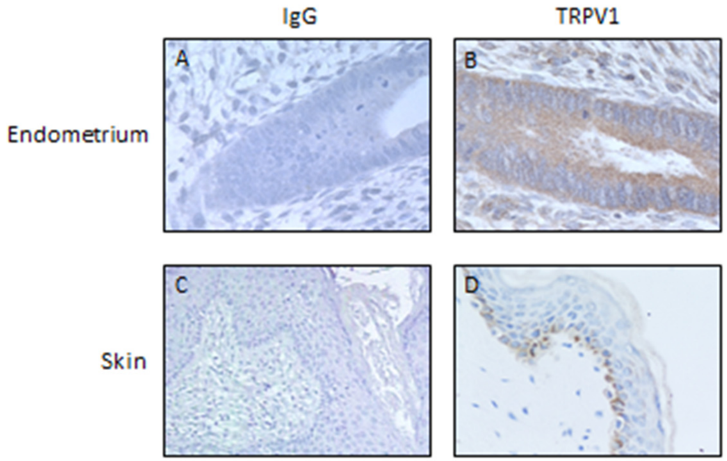

Supplemental figure demonstrating the specificity of the primary antibody to TRPV1. Images (A) and (B) show negative and positive staining pattern of TRPV1 antibody in proliferative phase endometrium and negative (C) and positive (D) staining for skin sections. Skin was selected as a positive control because TRPV1 is known to be present in parabasal cells of the epidermis. TRPV1 antibody was also evaluated in proliferative phase endometrium (A) and (B) and with equivalent concentrations of non-immune rabbit IgG, (A) and (C), which showed no staining (the negative control). Images for the endometrium were taken at 400x magnification, whilst images for the skin were taken at 100x and 200x magnification, respectively.
