# Supplementary material for: Expression, Distribution and Function of the Transient Receptor Potential Vanilloid Type 1 (TRPV1) in Endometrial Cancer
Source: Int J Mol Sci. 2025 Mar 27;26(7):3104. doi: 10.3390/ijms26073104 (PMC11988754; doi:10.3390/ijms26073104)
Supplement: Supplementary file 1 [file ijms-26-03104-s001.zip › Supplemental Table 1.pdf]

Supplemental Table 1. Description of the proteins examined, antibodies and dilutions used in the immunohistochemistry studies

Protein being examined	Name	Function	Antibody source	Host species	Optimised Primary Antibody Dilution	Reference
**TRPV1**	Transient receptor potential vanilloid 1	Ca^{2+} ion channel/(endo) vanilloid receptor	Alomone labs Cat # ACC-030	Rabbit	1 in 200	This publication
**GPR55**	Orphan G-protein coupled receptor 55	Non-classical (endo) cannabinoid/ Lysophosphatidylinositol receptor	Novus Biologicals, cat # NB110-55498	Rabbit	1 in 200	28
**CB1**	Cannabinoid receptor isoform 1	Classical, original isoform of cannabinoid receptor from brain that binds (endo)cannabinoids	Sigma Life Sciences, cat #C1108	Rabbit	1 in 500	32,13
**CB2**	Cannabinoid receptor isoform 2	Classical, original isoform of cannabinoid receptor from spleen that binds (endo)cannabinoids	Sigma Life Sciences, cat #C1358	Rabbit	1 in 150	32,13
**NAPE-PLD**	N-acylphosphatidylethanolamine-selective phospholipase D	Synthesis of *N*-acylethanolamines such as AEA	Sigma Life Sciences, cat # HPA024338	Rabbit	1 in 50	8
**FAAH**	Fatty acid amide hydrolase	Catabolism of *N*-acylethanolamines such as AEA	Alpha Diagnostics International, cat # FAAH11-s	Rabbit	1 in 2000	32,8
**Ki-67**	Proliferation Marker Protein Ki-67/ MiB-1	Proliferation marker associated with cellular mitosis	Novocastra, Cat # NCL-Ki-67-MM1)	Mouse	1 in 150	34
**BAX**	BCL2 Associated X, Apoptosis Regulator	Pro-apoptosis inducer through permeabilisation of mitochondrial membranes	Santa Cruz Biotechnology, Cat # SC-493	Rabbit	1 in 200	33
**Bcl-2**	Apoptosis Regulator BCL2	Proto-oncogene that prevents ROS activated cell death	Santa Cruz Biotechnology, Cat # SC-7382	Mouse	1 in 25	33

ROS=reactive oxidation species

8 Ayakannu, T., Taylor, A.H., Bari, M., Mastrangelo, N., Maccarrone, M. & Konje, J.C. (2019) Expression and function of the endocannabinoid modulating enzymes fatty acid amide hydrolase and N-acylphosphatidylethanolamine-specific phospholipase D in endometrial carcinoma. Front. Oncol. 9: 1363. [doi: 10.3389/fonc.2019.01363].

13 Ayakannu, T., Taylor, A.H. Konje, J.C. (2018) Cannabinoid receptor expression in estrogen-dependent and estrogen-independent endometrial cancer. J. Receptors Signal Transduct. 38: 385-392.

28 Ayakannu, T., Taylor, A.H., Konje, J.C. (2021) Expression of the putative cannabinoid receptor GPR55 is increased in endometrial carcinoma. Histochem Cell Biol 156: 449-460.

32. Habayeb, O.M., Taylor, A.H., Bell, S.C., Taylor, D.J. & Konje, J.C. (2008) Expression of the endocannabinoid system in human first trimester placenta and its role in trophoblast proliferation. Endocrinology 149: 5052-5060.
33 McLaren, J., Prentice, A., Charnock-Jones, D.S., Sharkey, A.M., Smith, S.K. (1997) Immunolocalization of the apoptosis regulating proteins Bcl-2 and Bax in human endometrium and isolated peritoneal macrophages in endometriosis. Hum. Reprod. 12: 146-152.
34 Taylor, A.H., Guzail, M. Wahab, M. Thompson, J.R. & Al-Azzawi, F. (2005) Quantitative histomorphometric analysis of gonadal steroid receptor distribution in the normal human endometrium through the menstrual cycle. Histochem. Cell Biol. 123: 463-474.
