# Supplementary material for: Expression, Distribution and Function of the Transient Receptor Potential Vanilloid Type 1 (TRPV1) in Endometrial Cancer
Source: Int J Mol Sci. 2025 Mar 27;26(7):3104. doi: 10.3390/ijms26073104 (PMC11988754; doi:10.3390/ijms26073104)
Supplement: Supplementary file 1 [file ijms-26-03104-s001.zip › Supplemental Tables 2 & 3.pdf]

Supplemental Table 2. Correlation analyses of the interactions between components of the endocannabinoid system in atrophic endometrium and endometrial cancer tissue.

		Endometrial Cancer									
		TRPV1	BAX	Bcl-2	BAX:Bcl-2	Ki-67	CB1	CB2	GPR55	NAPE-PLD	FAAH
Atrophic	TRPV1		-0.1425	0.1463	-0.1589	**-0.7550**	**-0.5379**	**0.5578**	**-0.4739**	**-0.6189**	**0.5163**
	BAX	-0.4769		0.082	0.142	0.373	**0.181**	-0.331	**0.417**	**-0.446**	0.440
	Bcl-2	-0.5388	-0.235		-0.251	-0.201	**-0.477**	-0.063	-0.219	**-0.440**	**-0.446**
	BAX:Bcl-2	0.3933	**0.374**	-0.839		0.195	0.093	0.072	-0.076	0.172	-0.087
	Ki-67	-0.0071	0.709	-0.247	0.631		**0.636**	**-0.563**	**0.469**	**0.708**	**-0.477**
	CB1	0.5409	-0.461	0.029	0.081	0.077		**-0.573**	**0.625**	**0.915**	**-0.849**
	CB2	-0.1696	0.609	-0.368	0.194	0.192	**-0.848**		-0.676	**-0.647**	0.253
	GPR55	0.5410	-0.778	0.274	-0.252	-0.304	0.276	-0.296		**0.679**	-0.344
	NAPE-PLD	-0.6899	0.455	0.182	0.031	0.224	0.056	-0.276	-0.765		**-0.644**
	FAAH	0.5901	-0.593	-0.148	0.199	-0.104	**0.927**	**-0.850**	0.279	0.046	

The data are presented as the Pearson correlation coefficient (r); inverse correlations are indicated with a minus sign placed before each number. Correlations that are significantly different to zero are shown in a red font and statistically significant relationships that are similar in both tissue types are shaded.

Supplemental Table 3. Correlation analyses of the interactions between components of the endocannabinoid system in Type 1 and Type 2 endometrial cancer tissue.

		Type 1 Endometrial Cancer									
		TRPV1	BAX	Bcl-2	BAX:Bcl-2	Ki-67	CB1	CB2	GPR55	NAPE-PLD	FAAH
Type 2 Endometrial Cancer	TRPV1		-0.32	-0.088	-0.209	**-0.773**	-0.422	**0.785**	**-0.586**	**-0.533**	**0.473**
	BAX	**0.669**		0.065	0.244	**0.560**	**0.614**	**-0.568**	**0.540**	**0.593**	**-0.669**
	Bcl-2	0.226	-0.190		-0.430	0.006	-0.361	0.074	-0.258	-0.308	0.320
	BAX:Bcl-2	0.295	**0.262**	-0.486		0.336	0.430	-0.353	0.217	0.359	-0.452
	Ki-67	0.187	0.241	-0.055	0.026		**0.687**	**-0.869**	**0.609**	**0.742**	**-0.752**
	CB1	**-0.938**	**-0.649**	-0.063	-0.329	-0.124		**-0.828**	**0.822**	**0.973**	**-0.980**
	CB2	**0.899**	**0.779**	0.106	0.106	0.041	**-0.870**		**-0.817**	**-0.893**	**0.862**
	GPR55	-0.192	0.184	-0.240	-0.240	0.105	0.222	0.011		**0.892**	**-0.817**
	NAPE-PLD	-0.344	-0.372	-0.153	-0.153	-0.395	0.397	-0.353	-0.302		**-0.967**
	FAAH	**0.972**	0.624	0.307	0.307	0.226	**-0.978**	**0.884**	-0.173	-0.429	

The data are presented as the Pearson correlation coefficient (r); inverse correlations are indicated with a minus sign placed before each number. Correlations that are significantly different to zero are shown in a red font and statistically significant relationships that are similar in both tissue types are shaded.
